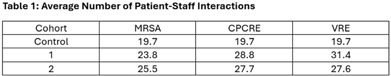# Is There a Correlation between Patient-Staff Interactions and MDRO Acquisition?

**DOI:** 10.1017/ash.2025.420

**Published:** 2025-09-24

**Authors:** Fadilah Zakaria, Aung Myat Oo, Maybelle Auw, Cawin Chan, Sean Whiteley, Edwin Philip Conceicao, Indumathi Venkatachalam, Xiang Ying Jean Sim

**Affiliations:** 1 National University of Singapore; 2 Singapore General Hospital; 3 AxoMem Pte Ltd; 4 Axomem Pte Ltd; 5 Axomem Pte Ltd; 6 Weien Chow; 7 Singapore General Hospital; 8 Singhealth

## Abstract

**Background:** Multi-drug resistant organisms (MDROs) are highly transmissible in hospital environments. Understanding the onward transmission of Methicillin-resistant Staphylococcus aurous (MRSA), Carbapenemase Producing Enterobacterales (CPE) and Vancomycin-resistant Enterococcus (VRE) is an important part of infection prevention. Patient-staff interactions can sometimes lead to MDRO acquisition. We aimed to investigate if patient-staff interactions were associated with MDRO acquisition in patients over 1 month in an inpatient tertiary hospital setting. **Methods:** During routine clinical care, staff will document in a patient’s electronic health records (EHR) upon interaction. Data regarding nursing and patient interactions were obtained extracting from “documents authored” and “vital signs” sections of the EHR. Hospital-Onset MDRO acquisition is defined as a positive detection of MDRO from a specimen collected on or after the third day of admission. The MDRO cohort was categorized by pathogen into two groups: before MDRO acquisition (cohort 1) and after MDRO acquisition (cohort 2). Patients with no known MDRO acquisition were selected as a control group. Average number of patient-staff interactions per day were calculated for each cohort. Descriptive statistics were employed and the appropriate post-hoc tests performed. Data analysis was performed using R (version 4.4.2) and RStudio IDE (version 2024.12.0). R package dunn.test (version 1.3.6) was used. **Results:** 8451 unique patients were included in the control group. Twenty-three MRSA and 35 VRE cases were included in cohort 1 and 2. For CRE, 41 cases were included in cohort 1 and 39 in cohort 2. Room type and location showed statistically significant relationship with (p < 0 .001) per-patient-daily average staff-inpatient interactions. The [MA3] average number of interactions per cohort can be seen in Table 1. Across all MDROs, the difference in patient-staff interactions between the cohort 2 and control group is significant (p < 0 .002). For VRE, the difference in patient-staff interactions between cohort 1 and control is significant (p=0.0015). **Conclusion:** Increased patient-staff interaction shows a correlation with VRE acquisition, but not for MRSA and CPCRE. [MA4] Further analysis is warranted to better understand the factors influencing the correlation between HCW interactions and MDRO acquisition.

**Figure:**